# Ultraviolet-Follow Curing-Mediated Extrusion Stabilization for Low-Yield-Stress Silicone Rubbers: From Die Swell Suppression to Dimensional Accuracy Enhancement

**DOI:** 10.3390/polym17060811

**Published:** 2025-03-19

**Authors:** Bing Liu, Baoji Ma

**Affiliations:** 1 School of Mechatronic Engineering, Xi’an Technological University, Xi’an 710021, China; 2 School of Mechanical and Electrical Engineering, Xi’an Polytechnic University, Xi’an 710048, China

**Keywords:** silicone rubber, UV curing, UFC, low yield stress, dimensional control

## Abstract

Direct ink writing (DIW) of low-yield-stress UV-curable silicone rubber faces challenges in maintaining dimensional accuracy due to post-extrusion swelling and viscoelastic deformation. This study proposes an ultraviolet-follow curing (UFC) method to enhance geometric precision by UV-follow curing (UFC) during deposition. The effects of UFC on filament dimensions—including width, length, cross-sectional geometry, and roundness—were systematically investigated. The mechanical properties of the printed structures were also evaluated to assess their functional integrity. The experimental results demonstrated that UFC effectively reduced extrusion swelling, achieving a filament-width deviation reduction of 22–81% and a filament-length deviation of 1.4–1.8% compared to conventional DIW. The printed rings exhibited improved roundness uniformity with reduced geometric fluctuations. Crucially, UFC enhances dimensional accuracy without compromising the mechanical performance of low-yield-stress silicones, providing a viable strategy for the high-precision additive manufacturing of soft elastomeric architectures.

## 1. Introduction

Silicone rubber is widely used in the wearable, biological, and aerospace fields due to its unique material properties, including high tensile elongation, biocompatibility, thermal and electrical insulation, chemical stability, and resistance to ultraviolet (UV) radiation [1,2]. Traditional silicone rubber manufacturing via molding faces limitations, such as the inability to fabricate highly complex or multi-scale high-precision structures. Three-dimensional printing, with its more flexible and economic advantages, has been widely used in silicone rubber. Various studies have been conducted in different fields [3,4,5], such as sensors [6,7,8], soft robotics [9], electronics [10], and foams [11], among others. For example, Codrin et al. [12] developed a 3D-printable organosilicon for dielectric elastomer actuators (DEAs). Y. Cheng et al. [13] achieved biocompatible soft robots with 3D printing technology, showing the suitability of 3D printing for complex designs. These studies collectively emphasize the precision, adaptability, production capabilities, and advantages of 3D printing for complex structures.

Among the various 3D printing methods, direct ink writing (DIW) stands out as a prominent approach for achieving 3D silicone printing [14]. The silicone material is squeezed out of the nozzle by applying pressure and is deposited in a layer, which is repeated layer by layer to form a three-dimensional part [15,16]. 

For successful DIW (direct ink writing) printing, first of all, the printing ink is required to have shear yield stress. When the material is extruded through the nozzle, the shear stress applied to the material enables the ink to pass through the nozzle smoothly. Secondly, the ink is required to have a high yield stress. After the ink passes through the nozzle, the shear yield stress disappears, and the high yield stress is restored. The high yield stress of the ink can ensure that the ink does not easily flow and diffuse during deposition, which helps to maintain the shape of the extruded filaments. Yield-stress DIW inks are mainly created by adding the appropriate fillers to liquid inks, such as fumed silica [17], polytetrafluoroethylene powder [18], liquid metal [19], graphene oxide [20], or gellan gum [21], and then the liquid ink is transformed into a yield-stress fluid. Other techniques for generating yield-stress behavior include particle charge stabilization and the addition of surfactants [22]. In addition, the capillary attraction between PDMS microbeads coated with PDMS liquid precursor has been used to generate yield stress [23]. Yield-stress materials can be rapidly transformed to maintain their shape, but they lack sufficient strength to support subsequent layers, handling, or functional use after printing. Therefore, a second TDS (time-dependent solidification) mechanism is needed to enhance the strength of green-printed parts. Thermal curing [24], chemical gelation [25], chemical cross-linking [26], and photo cross-linking [27] have all been proven to be secondary curing mechanisms. Similarly, materials with two time-varying curing mechanisms have also been demonstrated. Generally, the initial rapid curing is used to maintain the deposited shape of the material, while the second slower curing is used to improve the mechanical properties of the final part. Examples of this process are photo-curing followed by thermal curing [28] and solvent evaporation followed by oxidative cross-linking [29]. Thermal curing requires a long time of heating (>30 min), which easily leads to oxidation or deformation of the material [24], while chemical curing depends on the reaction rate (such as epoxy resin taking several hours), and the shape cannot be controlled in real time [26]. Although traditional photo-curing is fast, it requires overall irradiation and cannot inhibit local rheological relaxation.

Another approach is to make the printing ink undergo a curing reaction after deposition through the stimulation of an external device. Most of these methods involve modifying the DIW printer according to the special chemical components added to the ink, such as adding an ultraviolet light source [30] or using a two-component mixing extruder [31], a temperature-controlled extruder [32], or a temperature-controlled deposition plate [33]. In summary, thermal curing requires a high-temperature environment, which may limit the application of thermally sensitive materials. Due to the slow reaction speed, chemical curing is difficult to use to meet the needs of real-time molding. In contrast, photocuring is cured by instant cross-linking and is especially suitable for the high-precision printing of low-viscosity materials.

Through previous research, it can be concluded that the control of the dimensional accuracy of DIW printing silicone rubber mainlydepends on the improvement of printing ink viscosity and yield stress, as well as the improvement of printing equipment. Although these approaches have enhanced the dimensional accuracy of the printed filament, they impose certain requirements on the yield stress and viscoelasticity of the ink [34,35]. In some applications that require the use of low viscosity and low yield stress inks, such as electronic component manufacturing and microfluidic chips, dimensional accuracy cannot be guaranteed because ink with low yield stress will flow and creep after deposition due to the influence of its own weight [36,37]. This leads to considerable dimensional discrepancies in the formed filament and makes it difficult to achieve high-precision printing [38]. 

According to existing studies, traditional DIW printing can hardly form low-viscosity materials, thermal curing takes a long time to heat, and chemical curing is limited by the reaction speed, the long curing time brings difficulties to the molding of low-viscosity materials..

Therefore, we propose to print low-yield-stress inks using a UV-follow curing direct writing process (UFC). UFC achieves instant curing layer by layer through the following point light source. The curing speed (2 s) of UFC is significantly better than that of traditional DIW, thermal curing, and chemical curing methods. Its fast cross-linking characteristics are especially suitable for the precision molding of low-viscosity photosensitive materials. Table S1 shows a performance comparison of UFC and other curing methods.

A fast UV-curable silicone rubber with low yield stress was prepared as the printing ink. By comparing it with traditional DIW printing, the effects of UFC on the filament width, filament length, layer height, ring shape, and dimensional accuracy of hollow cylinders were studied. The effect of UFC on the mechanical properties of the product was also studied. The results showed that the UFC method ensured the forming of low-yield-stress inks and improved the dimensional accuracy of the printed samples.

## 2. Materials and Processes

### 2.1. Materials

In this research, all the printed specimens utilized the silicone rubber ink formulated by our research team as the UV-curable liquid silicone rubber ink. Our previous studies also reported in detail on the preparation and characterization of this ink [39]. This printing ink was based on the thiol-ene click reaction principle and could be UV-cured promptly. Vi-PDMS was employed as the base silicone rubber to provide end-vinyl double bonds, PDMS-SH offered thiol groups, and 2,2-dimethoxy-2-phenylacetone (DMPA) was adopted as the photoinitiator. DMPA disintegrated into free radicals under the effect of specific-wavelength UV light, which captured the hydrogen atoms on PDMS-SH to form thiol free radicals, which subsequently polymerized with the vinyl double bonds on PDMS-Vi to form alkyl free radicals. The alkyl free radical seized the hydrogen atoms on the thiol-containing compound to generate thiol free radicals anew, forming a cycle, which resulted in the continuous cross-linking of polymer molecules and the formation of a cross-linked network structure. The printing ink was made of Vi-PDMS, PDMS-SH, and DMPA in a certain proportion. The composition ratios are presented in Table S2.

**Figure 1:**
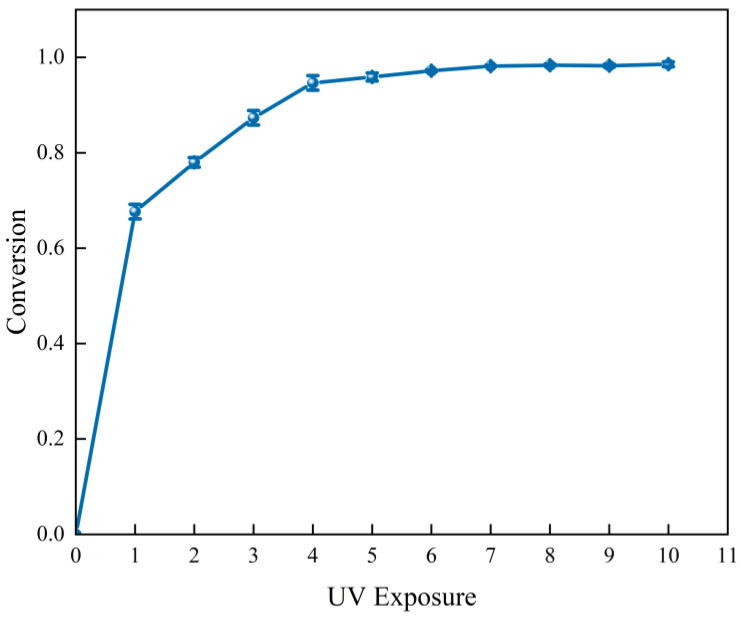
Conversion of UV silicon ink at different times.

The infrared (IR) spectra at various times under UV irradiation were collected using real-time IR (RT-IR) to characterize the conversion rate and curing speed of the silicone rubber (Figure 1). The liquid silicone rubber could rapidly be cured within 2 s under UV irradiation at a wavelength of 365 nm, an irradiation intensity of 40 W/cm^2^, and a distance of 20 mm. In order to allow the filler to better disperse in the ink system, this study selected polytetrafluoroethylene (PTFE) powder with a micron particle size as the thixotropic agent to adjust the viscosity and rheology of the printing ink. A certain amount of PTFE was added to the printing ink and stirred for 30 min to fully mix the LSR printing ink. The mixture was degassed in a vacuum chamber for 10 min to eliminate air bubbles.

### 2.2. Process of UFC

The printing system mainly comprised an XYZ 3D motion platform, a silicone rubber injector, a nozzle (a light-proof injector and nozzle were utilized in the actual experiments), a extrusion control module, a deposition platform, a UV lamp, a motion controller, a data acquisition system, and other related modules. The 3D motion platform was employed for the motion control of the substrate, the silicone rubber extruder, and the UV lamp. The X and Y axes adopted linear guides and synchronous belts for transmission, while the Z axis achieved up-and-down movement through a ball screw. The control module encompassed the control of the 3D motion platform and the silicone rubber extrusion module. To establish communication between the extrusion system and the motion platform, MAKERBASE was selected as the main control board of the system. The 3D model was processed by the host computer (Sympilify 3D) into G-code language, which was subsequently processed by the MAKERBASE main control chip to generate pulse signals for the stepping motor, controlling the motion platform to move along the designated trajectory. The extrusion control module was selected to have an air-driven extrusion approach. The UV lamp had a wavelength of 365 nm, featuring both point and surface light sources. The point light source had a light spot diameter of 3 mm, and the surface light source had a light spot area of 20 mm × 20 mm. Based on Auto CAD 2018 software, the model of the desired flexible device was constructed and exported as an STL binary digital format. Then, this file was imported into the Simplify 3D 5.1 slicing software for setting the printing parameters. The software converted the algorithm into a format recognizable by the device as G-code. Finally, the G-code file was imported into the lower computer of the printing device for printing. The extrusion control module controlled the air pressure in the injector to regulate the extrusion flow rate until a steady-state flow rate was attained.

**Figure 2:**
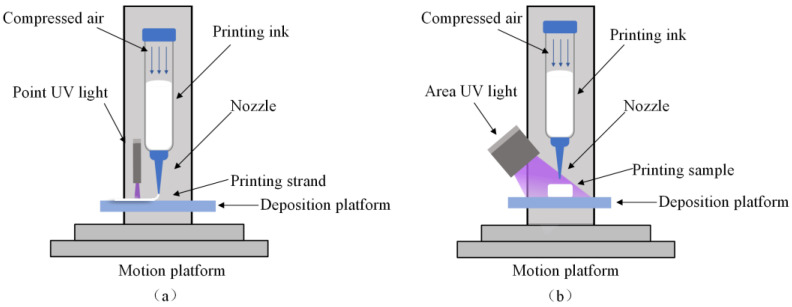
Three-dimensional printing and UV curing process with different methods: (**a**) UFC and (**b**) DIW.

The UFC system employed a point UV light source (Figure 2a), positioned 10 mm from the print nozzle to enable immediate curing post deposition. The point light source and the print head always moved at the same speed and trajectory. In this motion mode, the UV light source follows the printing nozzle to enable continuous curing during deposition.. During the printing process, the point UV light source was always on. The nozzle was extruded and deposited onto the substrate; the UV lamp then illuminated the newly deposited filament to cure it quickly. After completing one layer, the nozzle and UV lamp were lifted in the Z direction to build another layer.

The DIW printing method used an area UV light source (Figure 2b). The area light source did not need to move during the printing process, so the installation mode of the area light source on the printing system was fixed to ensure that the distance between the surface light source and the substrate did not exceed 50 mm. During the printing process, the area UV light source was not turned on. After the entire structure was printed, the light source was turned on and cured.

### 2.3. Rheological Property

Shear and oscillatory rheological tests were conducted on printing inks with varying proportions using rotary rheometers (MCR302, Anton Paar, Graz, Austria). The viscosity values of silicone rubber inks with different PTFE contents were measured at 25 °C within the shear rate range of 1–500 s^−1^. Stress scanning tests were performed within the shear stress range of 1–500 Pa at a frequency of 1 Hz. The dynamic modulus (G′ and G″) of silicone rubber inks with different PTFE contents was determined at room temperature.

### 2.4. Sample Preparation

To compare the influence of UFC on the dimensional accuracy of printing, silicone rubber straight lines 50 mm long were printed by both traditional DIW and the UFC described in Section 2.2. Low-viscosity and low-yield-stress inks were selected for the printing experiments. During printing, the UV light source was positioned at a distance of 10 mm from the extrusion nozzle and moved at the same speed as the nozzle, consistently following its trajectory. The silicone rubber wire cured immediately (within 2 s) upon contact with ultraviolet light. Under each set of printing parameters, two different methods—UFC and post-printing curing—were employed, respectively, to measure and compare the forming dimensions of two groups of lines under identical conditions, such as line width, length, and stack height. The extruded wire samples were captured using a CCD camera (i-SPEED 3, OLYMPUS, Japan) All experiments were repeated 5 times to verify the repeatability. Data are presented as mean ± standard deviation.

### 2.5. Statistical Analysis

The Welch-corrected independent sample t test was used to compare the print length difference between UFC and DIW in order to deal with the variance heterogeneity between groups. The normality of the data were verified by the Shapiro–Wilk test (*p* > 0.05), and the significance level was set to α = 0.05. The analysis was performed using the Python (3.13) SciPy library.

### 2.6. Mechanical Properties

The mechanical properties of the extrusion lines were evaluated using a universal testing machine and other specialized equipment. The tensile strength of the samples was determined using the UTM5305H universal tensile testing machine (DR-502A, Dongri Instruments Co., Ltd., Dongguan, China). Tensile strength tests for the rubber samples were conducted by ASTM D638-2003 [40], with a constant tensile rate of 100 mm/min. Before testing, the silicone rubber samples were shaped into dumbbell forms. Each sample type underwent 5 repetitions, and the reported value represented the average result.

## 3. Results and Discussion

### 3.1. Silicone Ink

**Figure 3:**
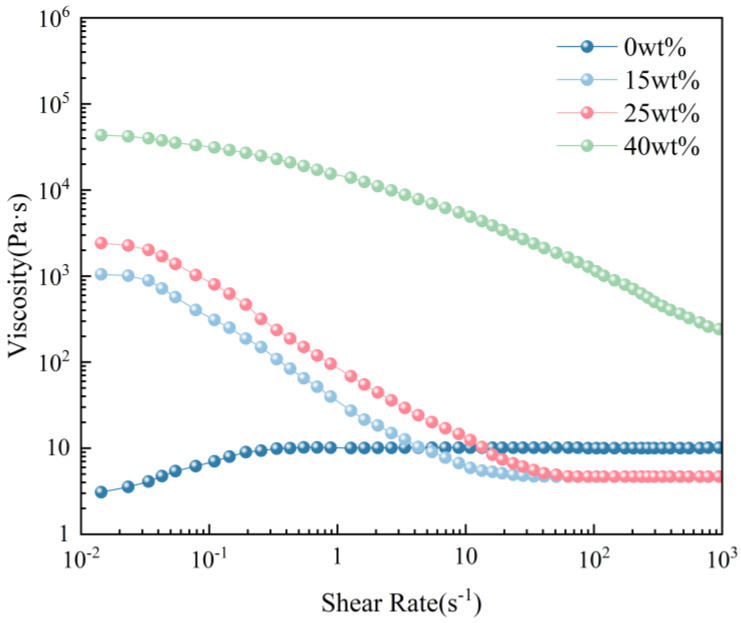
Viscosity (pa·s) versus shear rate (s^−1^) of printing ink combined with different PTFE contents.

**Figure 4:**
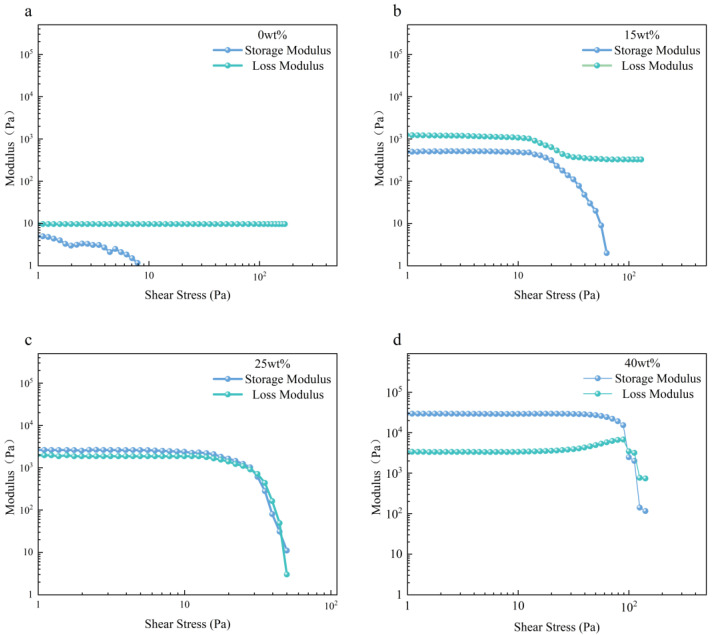
Oscillatory stress–sweep results for (**a**) ink with 0 wt% PTFE, (**b**) ink with 15 wt% PTFE, (**c**) ink with 25 wt% PTFE, and (**d**) ink with 40 wt% PTFE.

To investigate the impact of UFC on the printability and dimensional accuracy of low-yield-stress inks, printing ink with low yield stress was prepared by adjusting the mass fraction of polytetrafluoroethylene (PTFE). The rheological properties of silicone printing ink with different PTFE contents were experimentally obtained. Figure 3 illustrates the results from steady-state flow scans, while Figure 4 presents the results from oscillatory stress scans.

The storage modulus of the ink without PTFE content was always less than the loss modulus, and the loss modulus remained constant. The ink was in a fluid state. It could be observed that the apparent viscosity of the inks with added PTFE decreased as the shear rate increased, indicating shear-thinning behavior (Figure 4a). This was because the PDMS ink was a polymer, and the interaction between its molecular chains produced resistance to the flow, manifested as a high viscosity. When PTFE was added, the interaction between the two groups of particles and the molecular chain was kept relatively stable by the low shear rate. As the shear rate increased, the external shear force broke the entanglement between the PDMS molecular chains, and the interaction between PTFE and PDMS made the molecular chains slide more easily, resulting in a decrease in apparent viscosity and a shear-thinning property.

However, upon adding PTFE, both storage and loss moduli significantly increased. When 15 wt% PTFE was added, the storage modulus became lower than the loss modulus, resulting in a liquid behavior without shape retention force and poor molding performance (Figure 4b). Upon increasing the PTFE content beyond 25 wt%, although the storage modulus slightly surpassed the loss modulus, indicating an elastic response dominating over the others, creep still occurred at room temperature, making it unsuitable for traditional DIW and leading to poor accuracy (Figure 4c). Only when more than 40 wt% PTFE was added, the product exhibited a sufficiently high storage modulus (>104 pa·s) and shape retention. It could thus meet the requirements of traditional DIW (Figure 4d). This study focused on low-yield-stress inks. Based on these rheological property analyses for inks with different PTFE contents, two types were selected: one representing the flow properties (15 wt% PTFE) and another representing the creep properties (25 wt% PTFE). Both exemplified low-yield-stress inks lacking shape retention. The ratio of each component is shown in Table S3.

### 3.2. The Impact of UFC on the Precision of Filament Dimensions

**Figure 5:**
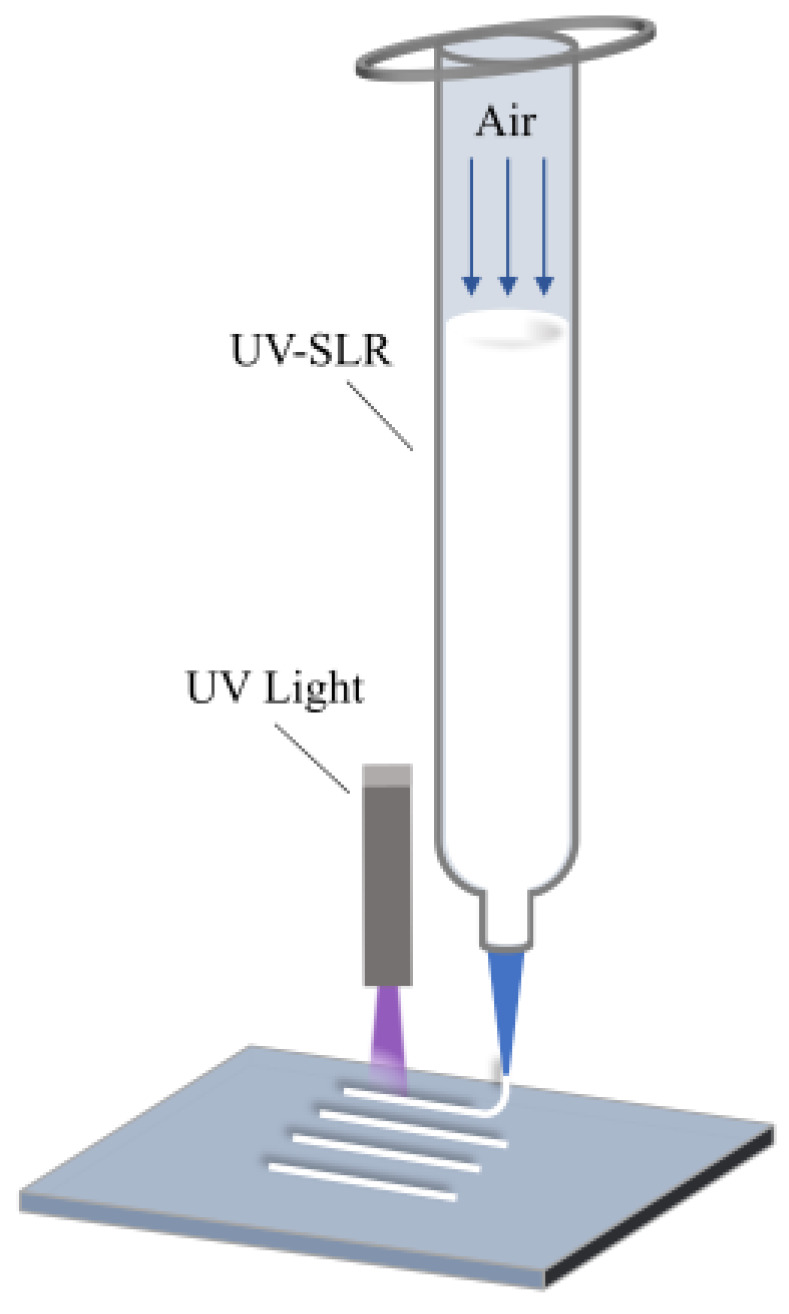
Schematic diagram of filament extrusion.

The filament dimensions in DIW are critical factors that significantly impact the overall structural accuracy. The precision and consistency of the filament size serve as the fundamental basis for ensuring accuracy. To assess the impact of the UFC printing method on dimensional accuracy by examining line dimensions, an experiment involving filament printing was conducted using a nozzle diameter of 0.84 mm, a nozzle movement speed of 1.0 mm/s, and an extrusion pressure of 0.2 MPa. The printing parameters are shown in Table S4. The extrusion process is shown in Figure 5. The CCD system was employed to analyze the filament dimensions.

#### 3.2.1. Single Filament

**Figure 6:**
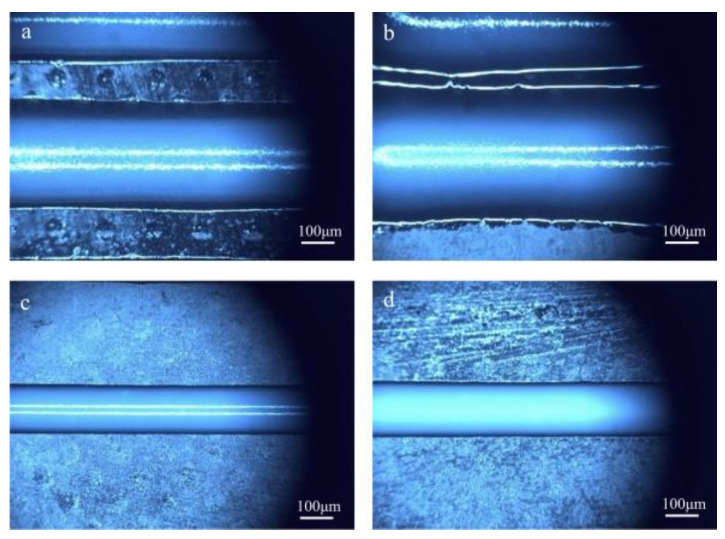
Images of the filament width of different inks by different printing methods: (**a**) PTFE 15 wt% by UFC; (**b**) PTFE 15 wt% by DIW; (**c**) PTFE 25 wt% by UFC; and (**d**) PTFE 25 wt% by DIW.

In this experiment, low-yield-stress inks with 15 wt% and 25 wt% PTFE mass fraction were selected, respectively. The printing parameters are shown in Table S3. Multiple silicone rubber filaments were printed using inks with varying rheological properties. Figure 6 illustrates the filaments extruded using different inks by UFC and DIW. The width of the filaments was measured after ultraviolet irradiation and curing. To assess the uniformity of the filament width, the standard deviation was calculated.

From the printing results of the 15 wt% PTFE ink (Figure 6b,d), it can be seen that the filament printed by DIW had uneven edges, poor uniformity, and a significantly larger filament width than the nozzle diameter. This was because the low viscosity of the ink caused it to flow like a fluid before curing, leading to collapsing, uneven edge formation, and large variations in filament width that exceeded the nozzle dimensions. By contrast, when using the UFC method, the edge flatness of the filament improved, with smaller molding diameters for the filaments, a reduced standard variance, and enhanced uniformity. For the 25 wt% PTFE ink, both methods resulted in relatively flat edges for the filaments (Figure 6c,d). Compared to the 15 wt% PTFE ink, the printing filament width was reduced, especially in the case of UFC printing, where the filament width closely approached the nozzle dimensions.

**Figure 7:**
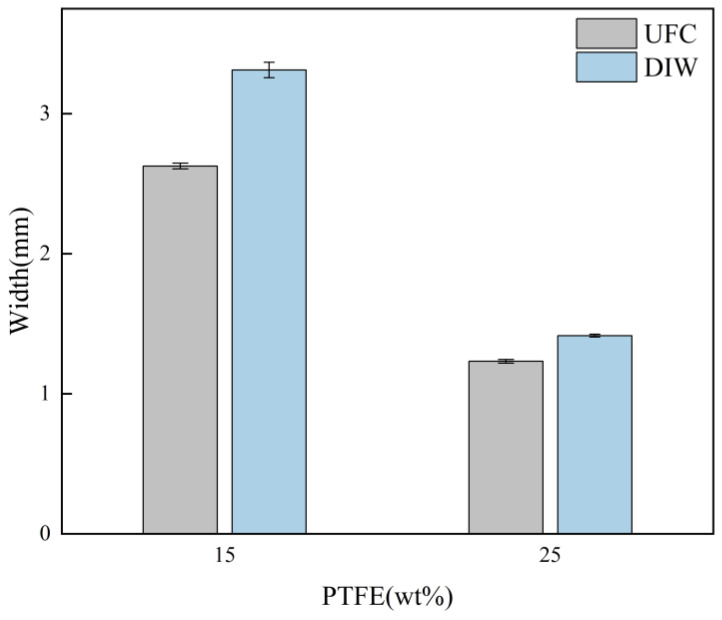
Filament width comparison between UFC and DIW for 15 wt% and 25 wt% PTFE inks (mean ± SD).

The calculated results of the measured filament width and standard variance are presented in Figure 7. The experimental findings indicate that the filament printed using UFC (Figure 6a,c) exhibited excellent overall edge flatness. Compared to the filament printed by traditional direct ink writing (DIW) (Figure 6b,d), the disparity between the filament and nozzle diameter was reduced. For inks with 15%PTFE content, the filament width of the UFC group was 2.63 ± 0.02 mm, which was significantly lower than that of the DIW group (3.31 ± 0.05 mm). For inks with 25%PTFE content, the filament width of the UFC group was 1.23 ± 0.01 mm, which was significantly lower than that of the DIW group (1.42 ± 0.01 mm). Moreover, the standard variance decreased, and the uniformity improved. As depicted in Figure 7, the width deviation measured for the 15 wt% PTFE ink was reduced by 81% compared to DIW, while that of the 25 wt% PTFE ink was reduced by 22%. These results suggest that the UFC printing method promotes the formation of low-viscoelasticity inks, thereby enhancing the dimensional accuracy of low-yield-stress inks with creep properties.

For 15 wt% PTFE inks, the mean value in the UFC group was significantly lower than that in the DIW group (t (5) = 25.98, *p* < 0.001), indicating that UFC significantly reduced the measured value at 15 wt% concentration. The mean value of 25 wt% PTFE inks in the UFC group was significantly lower than that in the DIW group (t (8) = 24.93, *p* < 0.001), indicating that UFC also significantly reduced the measured value at 25 wt% concentration.

This was because UFC inhibited the viscoelastic relaxation and gravity-driven flow of uncured inks by photoinitiating rapid cross-linking reactions that fixed the shape of filaments immediately after they were deposited. During curing, the rapid chain growth of the thiol-ene reaction (completed within 2 s) effectively reduced the ink’s relaxation time, thereby reducing expansion and deformation. In addition, the rigid support of the cured layer further limited the creep of subsequent sediments, ensuring geometric accuracy.

**Figure 8:**
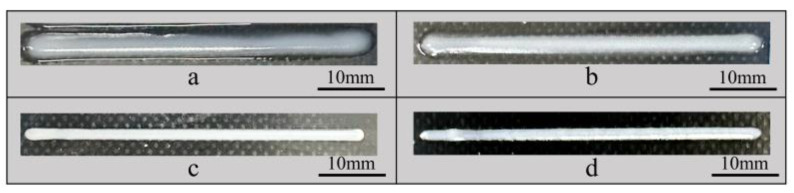
Images of the filament length of different inks by different printing methods: (**a**) 15 wt% PTFE by UFC; (**b**) 15 wt% PTFE by DIW; (**c**) 25 wt% PTFE by UFC; and (**d**) 25 wt% PTFE by DIW.

The printed lengths of the 50 mm filaments are shown in Figure 8. The lengths of the cured filaments were measured, and the experiment was repeated three times for each filament to obtain an average value. The deviation between the actual length and the design length was calculated: the smaller the deviation, the higher the length precision.

**Figure 9:**
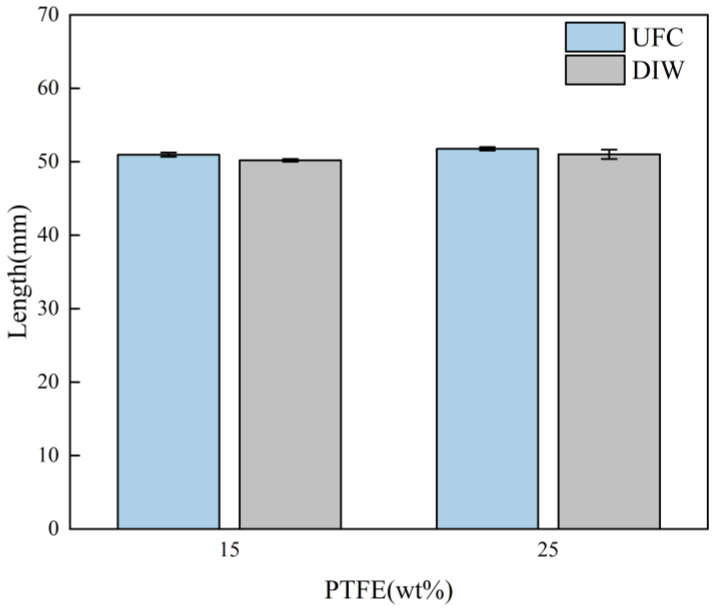
Filament length comparison between UFC and DIW for 15 wt% and 25 wt% PTFE inks (mean ± SD).

The deviation results between the printed length and the set length are shown in Figure 9. The experiments indicated that, for inks with low viscosity and fluid properties (15 wt% PTFE), the actual length of the filaments printed by UFC was 50.96 ± 0.28 mm, while the length printed by DIW was 51.77 ± 0.24 mm. The length deviation was significantly reduced in the UFC printing method. For inks with creep properties (25 wt% PTFE), the actual length of the filaments printed by the UFC method was 50.19 ± 0.18 mm, and the actual printed length by DIW was 51.02 ± 0.64 mm. The UFC printing method also reduced the length deviation accordingly. The length error of the 15 wt% PTFE ink filaments was reduced by 1.8% and that of the 25 wt% PTFE ink was reduced by 1.4%. This shows that the UFC printing method can improve dimensional accuracy in length, especially for inks with low viscosity and poor shaping ability.

For 15 wt% PTFE inks, the print length of the UFC group was significantly smaller than that of the DIW group (t (7) = 4.902, *p* < 0.001), indicating that UFC significantly reduced the length error of the 15 wt% PTFE inks. With the 25 wt% PTFE ink, the print length of UFC group was significantly better than that of the DIW group (t (4) = 2.790, *p* = 0.023), which verified the effectiveness of UFC on 25 wt% PTFE ink.

#### 3.2.2. Single Wall

In order to explore the influence of the UFC printing method on the dimensional accuracy of filament stacking, single-filament vertical-stacking printing was carried out to form a single wall. In this experiment, the printing ink with a PTFE content of 25 wt% was selected, the printing layer height was 0.3 mm, and the length of the printed single wall was 50 mm. Single walls with different stacking layer heights (single layer, three layers, five layers) were printed by UFC and DIW, respectively. The width, height, and stacking cross-sectional morphology of different layer heights were studied using CCD. The dimensions of the cured filaments were measured by CCD. For each measurement object, five points at the same distance were measured, each filament was tested three times, and the average value was taken. For different measured values, the standard deviation was calculated as a measure of uniformity.

**Figure 10:**
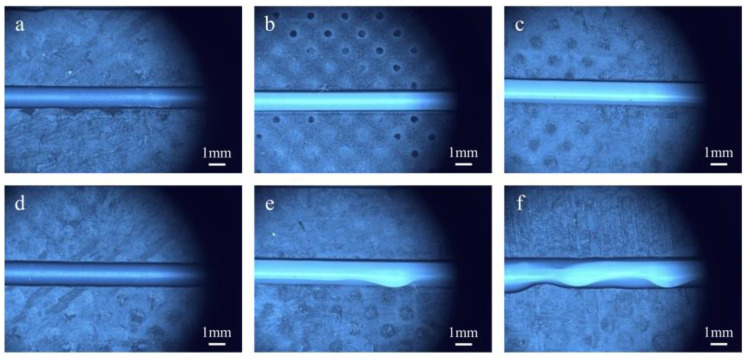
Images of the single-wall width of difference layers by different printing methods: (**a**) single layer by UFC; (**b**) three layers by UFC; (**c**) five layers by UFC; (**d**) single layer by DIW; (**e**) three layers by DIW; and (**f**) five layers by DIW.

**Figure 11:**
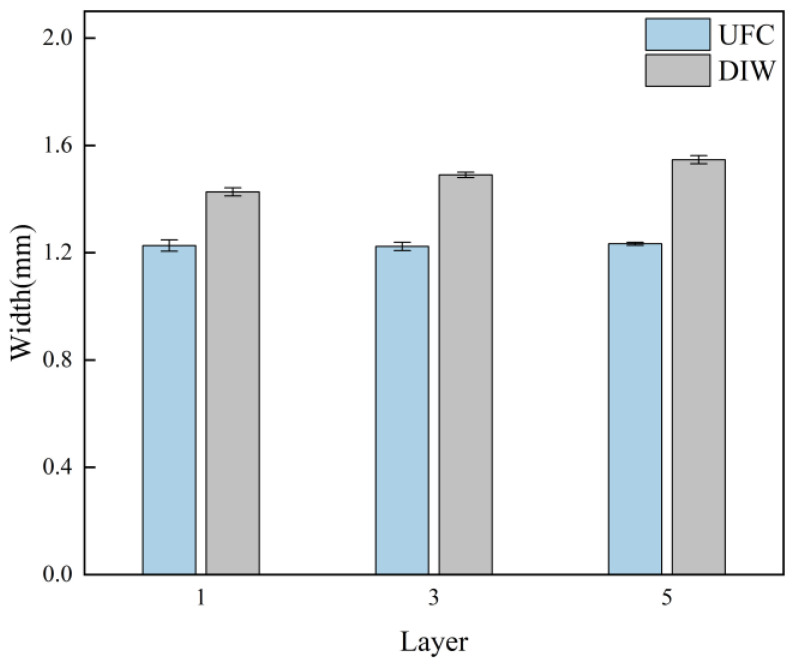
Single-wall width results for different amounts of printing layers under different printing methods (mean ± SD).

Figure 10 shows the top-view of the stacked single walls formed by different printing methods. The width of the stacked layers was measured. From the measurement results (Figure 11), it could be seen that, under the same printing parameters, the width of the filaments stacked at different layer heights printed by UFC was smaller than that of filaments printed by DIW. Evidently, UFC could promptly cure the filaments with low yield stress. During the extrusion process, there was no dimensional expansion, irregular shape, or influence on verticality caused by material flow and creep. This indicated that the UFC printing method could improve the dimensional accuracy of the line width of the stacked filaments.

Table S5 compares both the single-wall width variation (mean ± standard deviation) and the statistical significance between UFC and DIW across different numbers of stacking layers. Compared to DIW, the UFC method shows significantly enhanced efficacy across different numbers of stacking layers, as evidenced by the above analysis.

**Figure 12:**
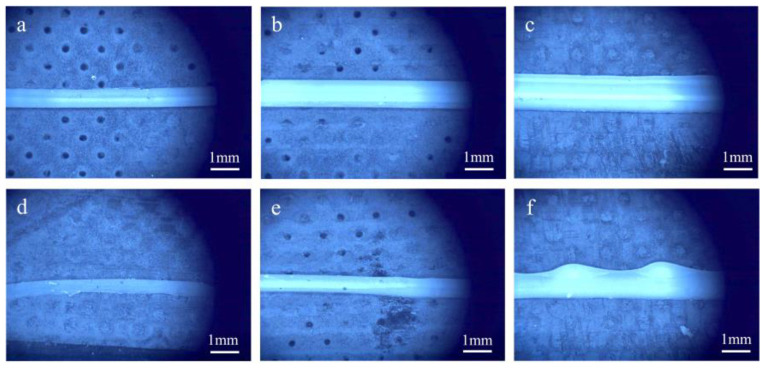
Images of the height of different amounts of layers by different printing methods: (**a**) single layer by UFC; (**b**) three layers by UFC; (**c**) five layers by UFC; (**d**) single layer by DIW; (**e**) three layers by DIW; and (**f**) five layers by DIW.

**Figure 13:**
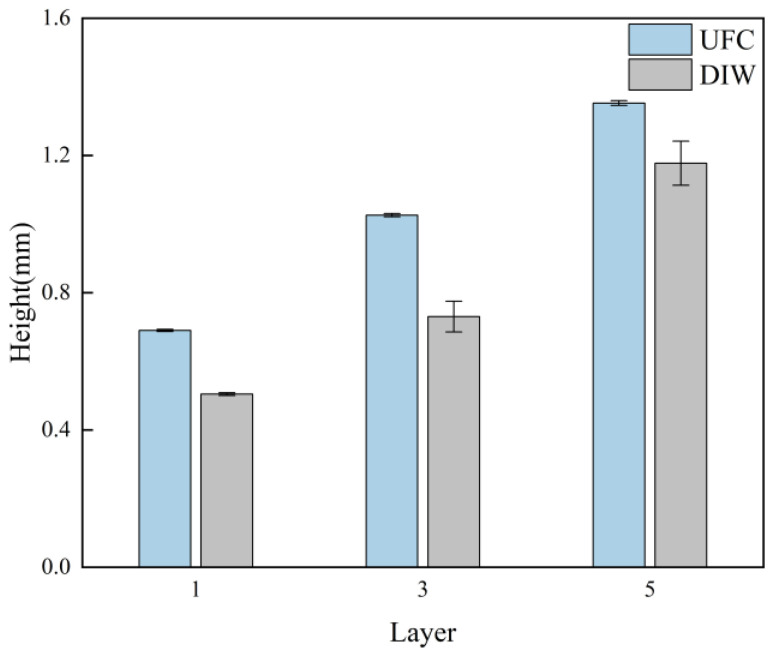
Single-wall height for different amounts of printing layers under different printing methods (mean ± SD).

Figure 12 depicts the side-view of the stacked single-filament walls fabricated via different printing methods, with the measurement focusing on the stacked layer’s height. The measurement and calculation outcomes are illustrated in Figure 13. Evidently, for the same number of stacking layers, the height of the filaments printed by UFC was, on the whole, greater than that of the filaments printed by DIW. Moreover, the deviation in layer height was diminished. The diameter error of the filaments with various amounts of stacked layers printed by UFC was consistently smaller than that of the samples printed by DIW. In other words, the errors resulting from the DIW printing method were higher than those from the UFC printing method. This result stemmed from UFC’s ability to rapidly cure the deposited filaments. The prompt curing of the preceding layer offered support for the subsequent layer, and, during the subsequent stacking and printing procedures, the preceding layer did not undergo creep or collapse caused by its own weight. Consequently, UFC effectively enhanced the dimensional accuracy of the stacking height of the filaments.

Table S6 compares the single-wall height difference (mean ± standard deviation) and statistical significance of UFC and DIW under different amounts of stacking layers. Through the above analysis, it can be clearly supported that the UFC method is significantly better than DIW under different amounts of stacking layers.

**Figure 14:**
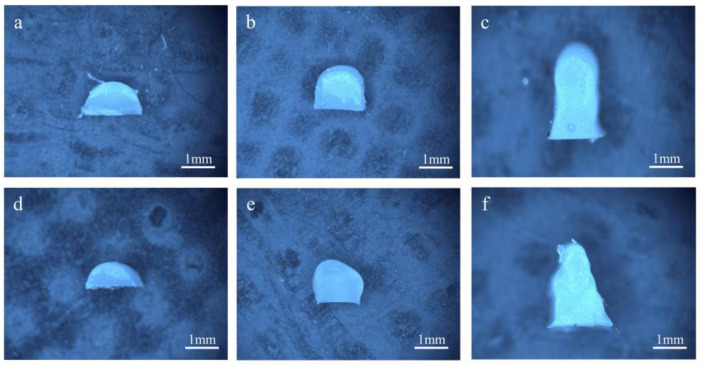
Cross-sectional images of different amounts of layers by different printing methods: (**a**) single layer by UFC; (**b**) three layers by UFC; (**c**) five layers by UFC; (**d**) single layer by DIW; (**e**) three layers by DIW; and (**f**) five layers by DIW.

Figure 14 shows the cross-sectional morphology diagrams of filament-diameter stacking at different printing layer heights under different printing processes. It can be observed that, for the filaments printed by DIW, the cross-sectional contour had an irregularly shaped boundary. The bottom layer experienced collapse and deformation, presenting an overall cross-section which was wider at the bottom and narrower at the top. Especially when stacking several layers, when printing the subsequent layer, the previous layer had not solidified yet. Due to the low-viscosity ink’s lack of shape retention, the preceding layer deformed under the weight of subsequently deposited layers, leading to significant distortion of the cross-sectional profile and dimensional accuracy.. From the cross-section of the filaments printed by UFC, it could be seen that the superposition of a subsequent layer hardly affected the shape of the previous layer. The bottom layer did not collapse, and the overall morphology was relatively good, with a certain degree of perpendicularity. Evidently, UFC could improve the cross-sectional morphology and dimensional accuracy of the single wall.

#### 3.2.3. Circular Ring

To explore the influence of the UFC printing method on extruded filament forming, single-filament ring printing was carried out. The printing parameters are shown in Table S7. With the other parameters kept constant, the morphological characteristics and filament diameters of the extruded rings made of low-yield-stress silicone rubber inks were analyzed and compared for different printing methods (the UFC printing method and the traditional printing method). In this experiment, inks with 15 wt% PTFE and 25 wt% PTFE were selected, respectively. The designed diameter of the ring was 10 mm. Based on different printing methods, multiple sets of silicone rubber rings were printed, and their roundness was studied using CCD.

**Figure 15:**
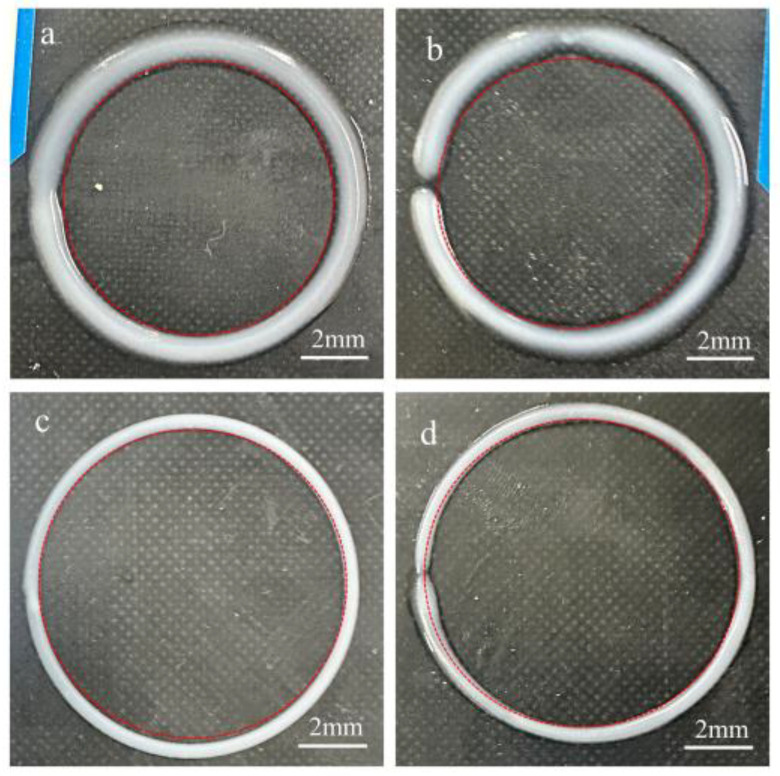
Morphology of circular rings of different inks by different printing methods: (**a**) 15 wt% PTFE by UFC; (**b**) 15 wt% PTFE by DIW; (**c**) 25 wt% PTFE by UFC; and (**d**) 25 wt% PTFE by DIW.

Figure 15 shows the ring-forming effect diagrams of inks with different yield stresses printed by two printing methods, UFC and traditional DIW. When using the DIW method (Figure 15b,d), phenomena such as extrusion filament collapse, poor uniformity, and inconsistent filament diameters occurred. In particular, when using the low-viscosity and low-yield-stress PTFE 15 wt% ink with fluidity, the dimensional error increased significantly, and the extruded filaments exhibited severe shape distortion, failing to retain the designed geometry.. When using the UFC printing method (Figure 15a,c), the morphology of the extruded filaments was improved. The extruded filament is uniform, the molding is good, and the phenomenon of broken filament is basically eliminated. Moreover, under the same printing method, the higher the yield stress of the ink, the better the formation. This indicated that UFC could significantly improve the shape-forming effect.

**Figure 16:**
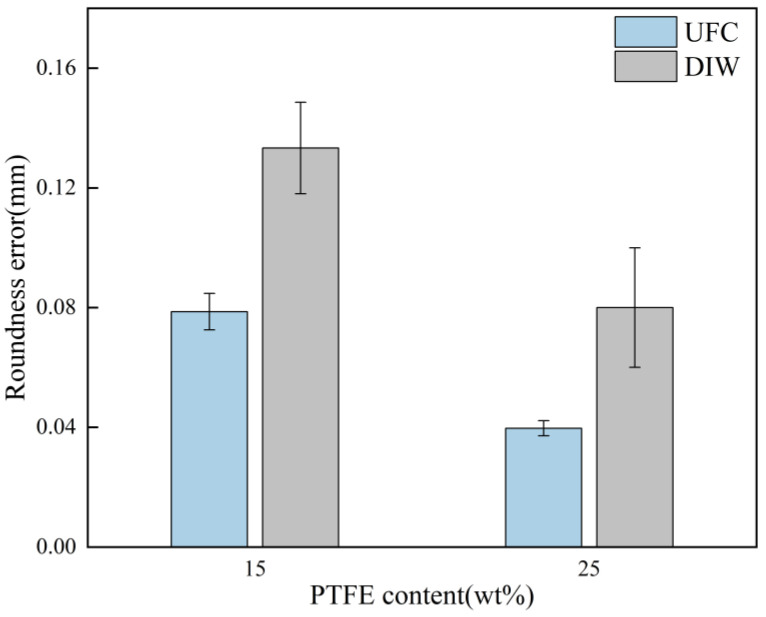
Section roundness error test results under different printing methods with different inks (15 wt% and 25 wt% PTFE) (mean ± SD).

To characterize the shape accuracy of the circles of different printed samples, roundness error tests were conducted on the printed samples, respectively. The test results are shown in Figure 16. The error range was between 0.04 and 0.12 mm. For the low-yield-stress ink with fluidity (15 wt% PTFE), the roundness error of the rings printed by the UFC process was reduced by 0.06. For the low-yield-stress ink with creep properties (25 wt% PTFE), the roundness error of the rings printed by the UFC process was reduced by 0.04. This verifies that UFC has better shape accuracy for low-yield-stress inks.

#### 3.2.4. Hollow Cylinder

The low-yield-stress silicone rubber ink (25 wt% PTFE) was used to print hollow thin-walled cylinders by the UFC and DIW printing methods. The nozzle diameter was 0.41 mm, and the printing parameters are shown in Table S8. The designed diameter of the hollow cylinder was 10 mm, and the height was 3 mm.

**Figure 17:**
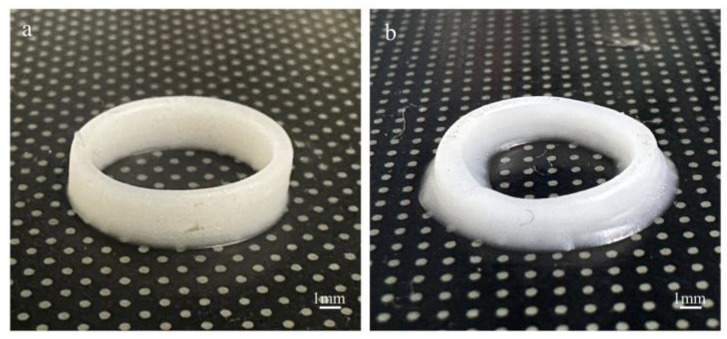
Morphologies of silicon ink architectures by different printing methods: (**a**) UFC and (**b**) DIW.

When using the UFC printing method (Figure 17a), the outer shape of the hollow cylinder was relatively complete, and the printed dimensions were consistent with the designed dimensions. The thin-walled cylinder formed by single-filament stacking could maintain its shape under the weight of subsequent layers. Moreover, the bonding between layers was excellent, without any cracks or delamination, presenting structural integrity However, when the same ink was printed by the DIW method (Figure 17b), collapse occurred, and there was a large deviation from the designed dimensions. This indicates that UFC can improve the formation of printed products made from low-yield-stress inks and ensure the accuracy of the printed dimensions.

### 3.3. The Impact of UFC on Mechanical Properties

**Figure 18:**
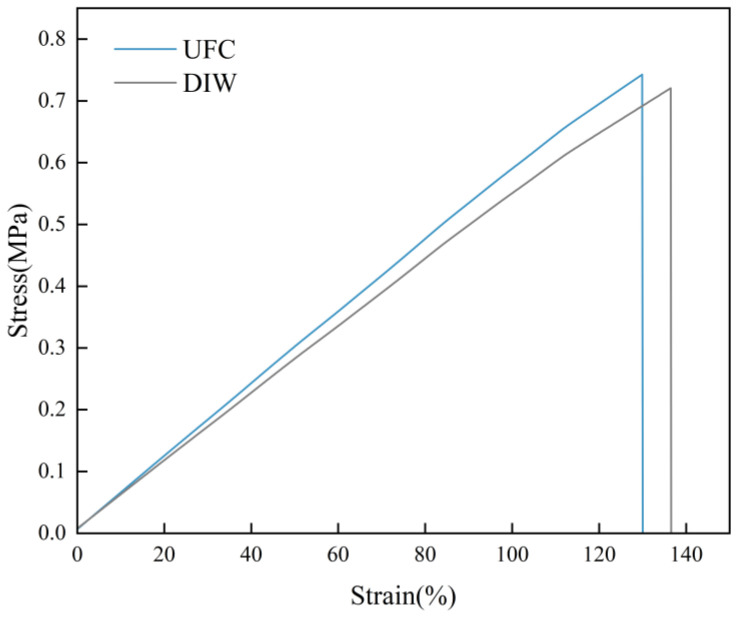
Tensile stress–strain curves of UV-cured samples.

Apart from dimensional accuracy, mechanical properties are also among the key factors in the application of silicone rubber products. The silicone rubber ink with a 25 wt% PTFE addition was selected to study the stress–strain curve of the parts printed by the UFC method. The nozzle diameter was 0.84 mm, and the printing parameters are shown in Table S8. Dumbbell-shaped specimens, which served as the mechanical test models, were printed using the UFC printing and curing method and the DIW printing and curing method. The mechanical properties of the dumbbell models were evaluated through uniaxial tensile tests. A universal tensile testing machine was used to conduct the tensile tests on the specimens. The stress–strain curves of the silicone rubber were obtained from the experiments (Figure 18). It could be seen that the tensile strength and elongation at break of the specimens printed by UFC were 0.74 MPa and 128%, respectively. For the specimens printed by DIW, the tensile strength and elongation at break were 0.72 MPa and 134%, respectively. Evidently, there was little difference in the tensile strength and elongation at break of the specimens formed by the two printing methods. This indicated that the UFC printing process did not reduce the mechanical properties of the printed specimens.

Voids between internal filaments primarily degraded the mechanical properties [15]. In the UFC printing method, a certain distance was maintained between the point light source and the extruder head, which meant that there was a short time interval between deposition and curing. Due to the specific creep property of the silicone rubber ink with the addition of 25 wt% PTFE, the filaments underwent short-term diffusion during this brief time interval after deposition and before curing. During the stacking process, the short-term diffusion of the filaments effectively filled the internal voids between the filaments, thus ensuring the mechanical properties of the printed parts.

The core advantage of UFC is its ability to accurately control low-yield-stress materials, especially for low-viscosity material systems which need to cure quickly and inhibit flow (such as flexible electronics, biocompatible packaging structures, etc.). However, this technology is not suitable for all material types. Table 1 summarizes the performance comparison between UFC and DIW. For high-viscosity or non-photosensitive materials (such as high-temperature-resistant polymers, epoxy matrix composites), traditional DIW or thermal curing methods have advantages. While UFC significantly improves dimensional accuracy, its compatibility with non-photosensitive materials remains a challenge, as discussed below. UFC needs specific photosensitive formulas (such as including an initiator). Point light source curing may increase equipment complexity. Multi-layer printing requires precise control of the light source distance; otherwise, it may cause over-curing. Therefore, the application of UFC needs to be targeted in combination with material characteristics and target scenarios to achieve the optimal balance of accuracy and performance.

## 4. Conclusions

This study demonstrated the efficacy of ultraviolet-follow curing direct ink writing (UFC-DIW) in achieving high-precision additive manufacturing of low-yield-stress UV-curable silicone rubbers. By incorporating polytetrafluoroethylene (PTFE) as a thixotropic modifier (15–25 wt%), two distinct UV silicone formulations with tailored fluidic and creep-dominant rheological behaviors were developed. Systematic comparisons between UFC and conventional DIW revealed critical advancements.

Regarding the dimensional fidelity enhancement, UFC reduced filament diameter deviations by 22–81% and length deviations by 1.4–1.8%, while maintaining superior cross-sectional morphology (roundness improvements of 0.04–0.06 mm).

Regarding structural stability, printed hollow cylinders exhibited minimized layer collapse, confirming the method’s capability to preserve geometric integrity in multi-layer architectures.

Regarding mechanical compatibility, tensile testing verified retained elastomeric properties, with UFC-processed specimens matching the intrinsic mechanical performance of bulk silicone.

The proposed UV-follow curing strategy establishes a paradigm for processing low-viscosity functional materials, addressing longstanding challenges in DIW of yield-stress-sensitive systems. This advancement expands the applicability of UV silicones in emerging fields requiring precision-structured soft matter, including magneto/electro-active composites and acoustic metamaterials. Furthermore, the methodology provides a scalable framework for manufacturing PDMS-derived architectures with submillimeter feature resolution, bridging critical gaps between rheological processability and microstructural control in soft robotics and flexible electronics. The UFC method holds promise for applications requiring both precision and soft material compatibility, such as flexible electronics and biomedical devices.

## Figures and Tables

**Table 1:** Performance comparison between UFC and DIW for different PTFE concentrations.

Method	Material Type	Curing Time	Width-Dimensional Error [%]	Length-Dimensional Error [%]	Mechanical Strength [MPa]
UFC	15 wt% PTFE	2 s	46.7	0.27	
	25 wt% PTFE	2 s	212.6	2.2	0.74
DIW	15 wt% PTFE	Depends on the printing time	68.5	2.07	
	25 wt% PTFE	Depends on the printing time	294.3	3.6	0.72

## Data Availability

Data are contained within the article and Supplementary Materials.

## Supplementary Materials

The following supporting information can be downloaded at https://www.mdpi.com/article/10.3390/polym17060811/s1, Table S1. Performance comparison between UFC and other curing methods; Table S2. The formula of PDMS ink; Table S3. The formula of ink; Table S4. The printing parameters of filament. Table S5. Comparison of signal-wall width (mean ± standard deviation) and statistical significance between UFC and DIW under different stacking layers; Table S6. Comparison of signal-wall height (mean ± standard deviation) and statistical significance between UFC and DIW under different stacking layers; Table S7. The printing parameters of the hollow cylinder. Table S8. The printing parameters of dumbbell specimens.
