# Supplementary material for: Ultraviolet-Follow Curing-Mediated Extrusion Stabilization for Low-Yield-Stress Silicone Rubbers: From Die Swell Suppression to Dimensional Accuracy Enhancement
Source: Polymers (Basel). 2025 Mar 19;17(6):811. doi: 10.3390/polym17060811 (PMC11946195; doi:10.3390/polym17060811)
Supplement: Supplementary file 1 [file polymers-17-00811-s001.zip › polymers-3533039-supplementary.pdf]

Supporting Information

Table S1. Performance comparison between UFC and other curing methods.

method	Curing time	Applicable material type	Data source
UFC	2s	Low viscosity photosensitive material	Experimental data of this study
Thermal curing	>30m	High viscosity thermal sensitive material	Zhang et al. [24]
Chemical curing	2h	High viscosity Epoxy resin materials	Nadgorny M et al. [26]

Table S2. The formula of PDMS ink.

Ink	Vi-PDMS [g]	PDMS-SH [g]	DMPA [g]
PDMS	100	3	2.16

Table S3. The formula of ink.

Printing Ink	Vi-PDMS [g]	PDMS-SH [g]	DMPA [g]	PTFE [g]
PTFE15wt%	100	3	2.16	18.56
PTFE25wt%	100	3	2.16	35.05

Table S4. The printing parameters of filament.

Ink	Printing method	Nozzle diameter [mm]	Speed [mm/s]	Extrusion pressure [MPa]	Wavelength [nm]	Light intensity [mW/cm^2]
PTFE15wt%	UFC	0.41	1	0.2	365	40
PTFE25wt%	DIW	0.41	1	0.2	365	40

Table S5. Comparison of signal-wall width (mean ± standard deviation) and statistical significance between UFC and DIW under different stacking layers.

Layer	Method	Width [mm]	t-value	Degree of freedon	P-value
1	UFC	1.226 ± 0.0152	22.60	8	<0.001
	DIW	1.428 ± 0.0130			
2	UFC	1.224 ± 0.0114	34.58	8	<0.001
	DIW	1.492 ± 0.0130			
3	UFC	1.230 ± 0.0071	32.24	5	<0.001
	DIW	1.546 ± 0.0207			

Table S6. Comparison of signal-wall height (mean ± standard deviation) and statistical significance between UFC and DIW under different stacking layers.

Layer	Method	Height [mm]	t-value	Degree of freedon	P-value
1	UFC	0.690 ± 0.002 mm	101.3	7	<0.001
	DIW	0.504 ± 0.003 mm			
2	UFC	1.026 ± 0.003 mm	20.57	4	<0.001
	DIW	0.729 ± 0.032 mm			
3	UFC	1.352 ± 0.005 mm	7.93	4	0.001
	DIW	1.177 ± 0.049 mm			

Table S7. The printing parameters of the hollow cylinder.

Ink	Printing method	Nozzle diameter [mm]	Speed [mm/s]	Extrusion pressure [MPa]	Wavelength [nm]	Light intensity [mW/cm2]
PTFE25wt%	UFC	0.41	1.2	0.22	365	40
PTFE25wt%	DIW	0.41	1.2	0.22	365	40

Table S8. The printing parameters of dumbbell specimens.

Ink	Printing method	Nozzle diameter [mm]	Speed [mm/s]	Extrusion pressure [MPa]	Wavelength [nm]	Light intensity [mW/cm2]
PTFE25wt%	UFC	0.41	1.3	0.22	365	40
PTFE25wt%	DIW	0.41	1.3	0.22	365	40
